# Important Considerations in the High-Dose Toxins Discussion

**DOI:** 10.1093/asjof/ojac031

**Published:** 2022-04-20

**Authors:** Jessica Brown, Julia E Herrmann, Conor J Gallagher

**Affiliations:** Revance Therapeutics, Inc., Nashville, TN, USA

We appreciate the comprehensive review of the aesthetic botulinum toxin A (BoNTA) high-dose (HD) studies^1^ as this topic has been of keen interest over the last few years and seems to have intensified of late. We would like to propose three considerations that we feel will add to and stimulate further discussion and analysis of these findings: (1) how can the significant inconsistencies in findings between studies be explained, (2) why are the findings of the abobotulinumtoxinA (ABO) HD study highly inconsistent with prior studies, and (3) time to return to baseline is not the most appropriate measure to estimate duration of effect.

The authors acknowledge that the currently approved BoNTA products are clinically similar, with limited evidence of differentiation. This observation is supported by the relative uniformity of results from their pivotal trials.^2-7^ Despite this, the HD studies yield quite different results in duration and peak efficacy. In the HD studies, similar results for median time to return to baseline were observed for incobotulinumtoxinA (INCO) and onabotulinumtoxinA (ONA), yet the ABO study showed a 40% longer result with a lower relative dose (2.5 times the on-label dose for ABO vs. up to 4 times the on-label dose for INCO and ONA).^8-10^ As both the formulation components and other study parameters were generally similar, it would be expected that the clinical performance would remain consistent between products.

Secondly, the HD results for the on-label dose and reconstitution of ABO are highly inconsistent with the registration studies. The reconstitution of the 50U ABO dose was the same as that utilized in the two published ABO glabellar lines (GL) pivotal trials. Therefore, one would expect that the results from the 50U control arm of the HD study would be similar to that found in the pivotal trials. The 2-point composite responder rate at week 4 in the HD study was 80% while the results from the pivotal trials ranged from 52% to 60%. Further, the reported duration for time to loss of none or mild of ABO 50U for GL in the pivotal studies ranged from 85 to 119 days compared to the ≈168 days reported for the same dose and reconstitution scheme for the 50U dose in the ABO HD study.^5,6,10^ Given these large discrepancies from the pivotal data, we find it hard to accept the findings from the ABO HD study. While this was not addressed in the article, we feel it is worthy of discussion.

Lastly, duration of effect in all three studies was reported as time to return to baseline (which can also be stated as time to loss of at least a 1 grade improvement in GL severity rating). However, the time to loss of none or mild wrinkle severity is the more commonly accepted and clinically meaningful duration endpoint and indeed was the key measure reported for the ABO and ONA pivotal trials.^2-7^ The authors point out that a 1-point improvement in a patient with severe GL is unlikely to be aligned with their patients’ goals of treatment, as it reflects the persistence of a minimally detectable improvement and not time spent in the desirable “treatment zone” of having none or mild lines. In the three HD studies, well over 50% of subjects had severe GL in every dose group studied.^8-10^ In patients with severe GL at baseline, returning to baseline requires traversing the “moderate” category and thus includes a period of time where their GL have fallen outside of the “treatment zone,” while still maintaining a rated 1 grade improvement. It would therefore be more informative for time to loss of a none or mild response be presented for the HD studies. In conclusion, beyond this insightful review, there remain some additional questions to be addressed in the consideration of the recently published/presented high-dose botulinum toxin studies.
